# Preparation and evaluation of decellularized epineurium as an anti-adhesive biofilm in peripheral nerve repair

**DOI:** 10.1093/rb/rbae054

**Published:** 2024-05-13

**Authors:** Xiao Li, Meihan Tao, Liang Quan, Hengtong Zhang, Yuan Xin, Xixi Wu, Xinyu Fang, Jun Fan, Xiaohong Tian, Xiaohong Wang, Lili Wen, Tianhao Yu, Qiang Ao

**Affiliations:** Department of Tissue Engineering, School of Intelligent Medicine, China Medical University, Shenyang, 110122, China; Department of Tissue Engineering, School of Intelligent Medicine, China Medical University, Shenyang, 110122, China; NMPA Key Laboratory for Quality Research and Control of Tissue Regenerative Biomaterial & Institute of Regulatory Science for Medical Device & National Engineering Research Center for Biomaterials, Sichuan University, Chengdu 610064, China; NMPA Key Laboratory for Quality Research and Control of Tissue Regenerative Biomaterial & Institute of Regulatory Science for Medical Device & National Engineering Research Center for Biomaterials, Sichuan University, Chengdu 610064, China; NMPA Key Laboratory for Quality Research and Control of Tissue Regenerative Biomaterial & Institute of Regulatory Science for Medical Device & National Engineering Research Center for Biomaterials, Sichuan University, Chengdu 610064, China; NMPA Key Laboratory for Quality Research and Control of Tissue Regenerative Biomaterial & Institute of Regulatory Science for Medical Device & National Engineering Research Center for Biomaterials, Sichuan University, Chengdu 610064, China; NMPA Key Laboratory for Quality Research and Control of Tissue Regenerative Biomaterial & Institute of Regulatory Science for Medical Device & National Engineering Research Center for Biomaterials, Sichuan University, Chengdu 610064, China; Department of Tissue Engineering, School of Intelligent Medicine, China Medical University, Shenyang, 110122, China; Department of Tissue Engineering, School of Intelligent Medicine, China Medical University, Shenyang, 110122, China; Department of Tissue Engineering, School of Intelligent Medicine, China Medical University, Shenyang, 110122, China; Department of Tissue Engineering, School of Intelligent Medicine, China Medical University, Shenyang, 110122, China; The VIP Department, School and Hospital of Stomatology, China Medical University, Liaoning Provincial Key Laboratory of Oral Diseases, Shenyang, 110001, China; Department of Tissue Engineering, School of Intelligent Medicine, China Medical University, Shenyang, 110122, China; NMPA Key Laboratory for Quality Research and Control of Tissue Regenerative Biomaterial & Institute of Regulatory Science for Medical Device & National Engineering Research Center for Biomaterials, Sichuan University, Chengdu 610064, China

**Keywords:** peripheral nerve, anastomotic adhesions, epineurium, decellularization, proteomics, anti-adhesion

## Abstract

Following peripheral nerve anastomosis, the anastomotic site is prone to adhesions with surrounding tissues, consequently impacting the effectiveness of nerve repair. This study explores the development and efficacy of a decellularized epineurium as an anti-adhesive biofilm in peripheral nerve repair. Firstly, the entire epineurium was extracted from fresh porcine sciatic nerves, followed by a decellularization process. The decellularization efficiency was then thoroughly assessed. Subsequently, the decellularized epineurium underwent proteomic analysis to determine the remaining bioactive components. To ensure biosafety, the decellularized epineurium underwent cytotoxicity assays, hemolysis tests, cell affinity assays, and assessments of the immune response following subcutaneous implantation. Finally, the functionality of the biofilm was determined using a sciatic nerve transection and anastomosis model in rats. The result indicated that the decellularization process effectively removed cellular components from the epineurium while preserving a number of bioactive molecules, and this decellularized epineurium was effective in preventing adhesion while promoting nerve repairment and functional recovery. In conclusion, the decellularized epineurium represents a novel and promising anti-adhesion biofilm for enhancing surgical outcomes of peripheral nerve repair.

## Graphical Abstract

**Figure:**
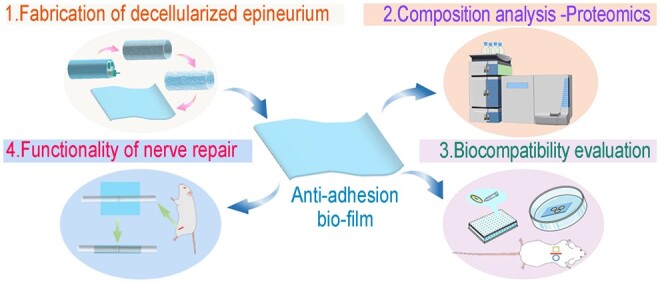

## Introduction

Peripheral nerve injury (PNI) is a typical disease leading to motor, sensory and trophic impairment in innervated region [1]. Annually, over 1 million individuals globally are afflicted with PNI, with approximately 300 000–500 000 cases reported in China, constituting 2.8% of all trauma cases [2], which imposes a significant medical and economic burden on society. Recently, due to the fast development of microsurgical techniques, the quality of nerve anastomosis has been greatly improved [3, 4]. Nevertheless, a significant challenge associated with neuroanastomosis is the development of fibroblast scars at the neuroanastomosis site [5]. Even in adequately repaired nerves, roughly 50% of the regenerated axons might extend into tissue scarring, potentially resulting in a localized neuroma and impeding axon regeneration toward its intended target [6]. Consequently, regenerative nerve function often remains unsatisfactory. Therefore, preventing nerve adhesion and providing a good anastomosing microenvironment is of great significance in promoting nerve regeneration and repair.

In recent years, a series of materials have been developed to avoid site adherence following nerve anastomosis, which are mainly polymer films [7–9]. While these materials offer some resistance to adhesion, they exhibits low biocompatibility due to the lack of bioactive components. This has led to an increasing shift toward natural materials like chitosan [10–12] and collagen [13, 14]. Their enhanced biocompatibility is conducive to adhesion of cells and proliferation, crucial for tissue engineering applications. However, natural materials are not without their drawbacks. One significant issue is the fast degradation rate, which cannot match the rate of nerve repair. Additionally, the variability and inconsistency in their physical and chemical properties, which can arise from differences in their biological sources, can lead to challenges in standardizing and replicating results. Research indicates that when it comes to tissue rebuilding, extracellular matrix (ECM) components—especially those sourced from tissues specific to a certain site—work better than synthetic or non-specific tissue-derived materials [15–17]. ECM is a complex, multidimensional structural network present in a variety of tissues. It is essential for tissue remodeling, organization, and control over cellular functions [18]. Collagen, laminin (LN), proteoglycans (including glycosaminoglycans (GAGs)), elastic fibers and elastin, fibronectin (FN), and other glycoproteins are some of its primary constituents. The ECM serves as a communication network between cells within organs and tissues, transmitting biochemical and mechanical signals. During cellular migration, it acts as a scaffold, providing structural support and potential attachment points for cells [19–21]. Decellularized ECM (dECM) refers to a biological material derived from tissues or organs of humans or animals through decellularization techniques, which eliminate immunogenic cellular components, mostly retaining the ECM as their primary component [22, 23], Therefore, dECM is under the spotlight. Ting Li *et al*. [24] prepared the nerve anti-adhesion membrane after the decellularization of the porcine sciatic nerve and carried out studies *in vitro* and *in vivo*, respectively, which confirmed this anti-adhesion membrane could effectively avoid the invasion of tissues adjacent into the site of anastomosis, lessen adherence of tissues while promoting regeneration of nerves. However, this kind of membrane has a low mechanical property and rapid degradation *in vivo*, which makes it difficult to match the speed of nerve repair and has certain limitations. The epineurium is a natural biological scaffold extracted from nerve tissue that has been specially processed to remove cellular components but retain the intact structure of the ECM. It has good biocompatibility and bioactivity, and a suitable degradation rate *in vivo*, which will minimize the risk of postoperative adhesion as well as offer the best possible support structure for nerve regeneration.

The present study explores the development and efficacy of decellularized epineurium (DEP) as an anti-adhesive biofilm, aiming to address the challenges of peripheral nerve repair with an innovative, biomimetic approach.

## Materials and methods

### Fabrication process of epineurium

Epineurium was prepared following the guidelines outlined in the ISO 22442-2 standard. Fresh nerves of the lower limbs of crossbred porcine gained from the market were removed from external fibrous tissue, blood and fatty tissue, and then cut into 5 cm segments. Following freezing at −80°C for 2 h, the sciatic nerves were lyophilized in a freeze drier for 6 h. The dried nerves were temporarily submerged in PBS for 30 s for sufficient moisturization of the epineurium. Subsequently, microscopic scissors and forceps were used to delicately separate the epineurium from the nerves. The isolated epineurium was then sliced into small pieces of 2 cm by 2 cm and repeatedly cleaned in PBS adding 1% antibiotics. Two sections of materials were separated: half lyophilized and kept for the further program; the other was put in PBS for additional decellularized treatment.

### Process of the epineurium decellularization

The epineurium was first immersed for 6 h, with three fluid changes. Following this, submerged in 3% Triton X-100 solution (Sigma, USA) ∼12 h. Subsequent to this immersion, three 10-min washes with deionized water were performed on the samples. They were then placed in sodium deoxycholate solution (4% (w/v)) (Sigma, USA) ∼24 h, after which there were three further 10-min rinses with deionized water. These treatments were all carried out in a shaking incubator with a 120 rpm setting at ambient temperature. Ultimately, the DEP was placed at 4°C for later use after being moved to a PBS solution containing 1% antibiotics.

### Assessing the efficacy of DEP

To evaluate the effectiveness of DEP, both DEP and fresh epineurium (FEP) samples underwent H&E staining, along with DNA analysis. Following fixation in a 4% (w/v) paraformaldehyde solution for 24 h, after being dehydrated in ethanol, FEP and DEP were embedded in paraffin and sectioned into slices that were 4 μm. H&E staining was used for the observation of nuclei. Furthermore, a genomic DNA extraction kit (Tiangen, China) was used to extract DNA in accordance with the instructions provided by the manufacturer. In order to determine the quantity of DNA that was still present in the DEP, the extracted DNA was measured using an absorbance spectrophotometer (Bio Drop Duo, UK). Utilizing 1% agarose gel electrophoresis, DNA fragments were isolated in order to conduct a qualitative analysis.

### GAGs analysis

By employing the dimethylmethylene blue method (GenMed Scientific Inc, USA), the GAGs content was evaluated and determined in accordance with the instructions provided by the manufacturer.

### Proteomic analysis

Following the iFASP method's preparation of protein samples and determination of their protein concentration, the samples underwent enzymatic digestion. The results of enzymatic digestion were subjected to RP-ESI-MS/MS analysis, employing an Orbitrap Fusion Lumos mass spectrometry equipment in conjunction with an Easy-nano LC 1200 liquid phase apparatus. Positive ion scanning mode was used for mass spectrometry, and data-dependent mode was used for the acquisition of secondary mass spectrometry. Utilizing software called Xcalibur 1.4 (Thermo Fisher, USA), system control and data capture were handled. Retrieval of cellular proteome mass spectrometry data for label-free quantitative samples was achieved through Maxquant (version 1.6.5.0), with the database sourced from the UniProt website (Sus Scrofa domestic’s whole proteome of domestic porcine origin). The LFQ algorithm was employed for protein quantification, with the ‘match between runs’ function enabled. The target-decoy approach was utilized to set the false discovery rate at 1% for both protein and peptide levels.

Bioinformatics analysis encompassed protein enrichment and the gene ontology (GO) analysis, covering cellular composition, biological processes, and molecular functions based on the protein data.

### Scanning electron microscopy observation of the DEP

Utilizing scanning electron microscopy (SEM), the microscopic structure of the DEP was observed. After fixing the material for 6 h at 4°C with 2.5% glutaraldehyde (Sigma, USA), they were rinsed three times for 5 min each with PBS. Then utilizing an ethanol gradient to do successive dehydration, three rinses with deionized water were conducted. Samples were then dried in a vacuum freeze dryer for an entire night. A 10 kV accelerating voltage was used for observing the microstructure with a tungsten SEM (TESCAN, Czech).

### Water absorption analysis

To assess the water uptake of DEP, studies with water absorption were conducted. In order to achieve a consistent dry mass, the materials (*n* = 5) were lyophilized for 12 h after being submerged in PBS for 24 h. After lyophilization, the materials were immersed in PBS for a whole day at 4°C in order to attain a stable weight upon absorption. The weight after absorption was subtracted from the dry weight to calculate the water weight. By dividing the water weight by the dry weight, the water absorption rate was determined.

### Evaluation of the DEP’s biosafety

#### Cytotoxicity assays

The Cell Counting Kit-8 (CCK-8) assay was used to assess the cytotoxicity of DEP. The DEP was sterilized with 75% alcohol for ∼2 h, washed three times with sterile PBS, and then submerged in sterile PBS to be used. Extracts were produced by incubating the sterilized DEP for 24 h at 37°C at a concentration of 6 cm³/ml in a medium adding serum. Positive controls (DMSO), negative controls (polyethylene), and blank controls (complete medium) were prepared accordingly. In 96-well plates, RSC-96 cells were planted with a density of 3 × 10^3^ each well. Upon completion of the incubation period of 24 h, the culture medium was changed and 100 μl of either the positive, negative or blank control extract (DEP) was added into every well. Afterwards further incubation of 1, 3 and 5 days, 10 μl of the CCK-8 solution was put into every well, and then the incubation time was extended to 1 h. After that, an enzyme-linked measuring device was employed to ascertain absorbance at 450 nm. The OD value was then used to compute cell viability, which was used to assess DEP cytotoxicity.

#### Hemolysis test

Blood was extracted from Sprague–Dawley rats in order to get cleaned erythrocytes. The plasma was extracted from the blood by centrifuging it for ten minutes at 4°C at 1000 rpm. Erythrocytes, usually leftover red blood cells, were suspended in saline solution. Three to five times was the washing procedure repeated, up to when the supernatant turned translucent and colorless. Following this, the blood cells were suspended in 2% (v/v) saline.

To obtain DEP extracts, the DEP was sterilized with 75% alcohol ∼2 h, washed three times with sterile PBS, and then immersed into saline with a concentration of 6 cm^2^/ml and incubated over 24 h at 37°C. The supernatant that resulted was gathered. Three centrifuge tubes were then filled with 0.5 ml of a 2% erythrocyte suspension, 1 ml of distilled water, DEP extract and saline (as a positive control and negative control, respectively). The supernatant was removed after hemolysis was visually inspected, and an enzyme-linked measuring device was employed to ascertain the absorbance at 545 nm. The hemolysis rate (HR) (%) was used to indicate the extent of hemolysis, calculated using the formula provided below:

(1) HR(%)=[(ODtest−ODnagative)/(ODpositive−ODnegative)]×100%

#### Cell affinity assay

The cell affinity of RSC-96 cells on DEP was observed by SEM. (1) Sample preparation: The materials were sliced into 1 cm by 1 cm pieces, sterilized with 75% alcohol for ∼2 h, three times with sterile PBS washed, and then submerged in sterile PBS to be used. (2) The cell suspension was added at a cell count of 1 × 10^5^ per well, then cultured in an incubator after the materials were progressively put into the 24-well plate. (3) After 3 days of incubation, the materials were taken out and repeated three times for 5–10 min each time, followed by an overnight soak in 2.5% glutaraldehyde. (4) Then washed three times for 5–10 min each time with PBS solution, and placed in an ultra-clean bench at room temperature to allow them to dry. (5) Observe the changes in cell morphology on the surface by SEM.

In addition, cell suspensions containing 1 × 10^5^ RSC-96 were cultured in DEP extract. After 3 days, after fixation with 4% paraformaldehyde, the samples were stained with AbFluor 488 Reagent (BMD0082; Abbkine, USA) and then viewed by confocal microscopy.

#### Immune reaction to implanting subcutaneously

Experiments on animals adhered strictly to the approved regulations by the Experimental Animal Ethics Committee of China Medical University (CMU2019192). Animals were divided into two groups: DEP and decellularized nerve repair membrane (DNRM), respectively. The DEP and DNRM were cut to the size of 1 cm × 0.5 cm, sterilized with 75% alcohol ∼2 h, three times with sterile PBS washed, and then submerged in sterile PBS to be used. SD rats were anesthetized with sodium pentobarbital (using a ratio of 30 mg/kg), and then dorsal skin preparation and disinfection were done. A 1.5-cm longitudinal incision was created along the rat’s back’s midline. The material was inserted into a subcutaneous capsule that was created by blunt dissection and was situated 10 mm to the left of the midline. In the same way, a subcutaneous capsule was prepared 10 mm to the right of the midline, but no material was implanted as a control group (Figure 5D). The incision was sutured. After the rats had awakened from anesthesia, they were returned to the cage for normal rearing.

Samples were collected at 1, 4 and 6 weeks post-implantation. Following humane euthanasia, the implanted material with surrounding tissue was obtained. To assess the immunological response to the implanted materials, immunofluorescence and H&E staining were used. Anti-CD11b antibody (diluted 1:100; Abcam, USA) was used to identify neutrophils, while anti-CD68 antibody (diluted 1:100; Abcam, USA) was used to identify macrophages. Images of stained sections were viewed by a light microscope, and for analysis, the fields with top, bottom, left, right, and center of vision were selected. The CD68 and CD11b positive area ratios were calculated using ImageJ software.

### Functionalities evaluated *in vivo*

#### Surgical protocols

Rats were fasted and dehydrated for 8 h. Animal experimentation adhered strictly to the approved regulations by the Experimental Animal Ethics Committee of China Medical University (CMU2019192). All materials were sterilized with alcohol. In brief, materials were immersed in 75% alcohol for 2 h, then washed three times with sterile PBS, and finally stored in sterile PBS for later use. All surgical operations were performed on the right leg. The right leg was weighed and given an intraperitoneal injection of sodium pentobarbital (1% w/v) at a dose of 40 mg/kg to induce anesthesia, after which the limbs were fixed on surgical plates. The surgical area was subjected to a series of operations such as hair removal, skin preparation, disinfection and toweling. A 20-mm incision was made by longitudinal dissection in the posterior lateral femur, and blunt separation was performed along the course of the muscle fibers till the sciatic nerve is exposed. Then the nerve was cut. 8-0 nylon microsutures were used to close the epineurium both distally and proximally to the nerve terminal. The DEP and DNRM were wrapped around the anastomosis, respectively. The sciatic nerve was cut for end-to-end anastomosis without the use of materials wrapping in the control group. Afterwards, a 4-0 nylon suture was used to suture together the muscle and skin, and finally, a little penicillin powder was applied to the wound. After awakening, the rats were put back in the cage to be raised normally.

#### Claw-spread reflex examination

At the fourth and eighth weeks following surgery, the claw-spread reflex was tested to assess the functionality of regenerating nerves. Using the following standards, nerve function recovery was categorized into grades A, B or C: Grade A indicates that the rat reacts to acupuncture and spreads the claws; Grade B indicates a response to acupuncture with no claw spreading; Grade C indicates no response to acupuncture. A double-blind method was used for the statistics.

#### Observation of tissue adhesion at nerve anastomosis

At 4 and 8 weeks following surgery, the adhesion with adjacent tissues was assessed at fourth and eighth weeks post-surgery using a grading system comprising three levels: Grade 0 indicates no adhesion; Grade 1 indicates slight adhesion, where the anastomosis site can be separated with ease; Grade 2 indicates tight adhesion, making separation difficult. A double-blind method was used for the statistics.

#### Sciatic nerve function index

The CatWalk XT gait analysis system was purchased from Noldus Corporation. Briefly, the instrument has a single-channel system, when the rat passes through the channel, there is a camera at the bottom of the channel that can record each step of the rat and transfer the data into the system software, after the CatWalk XT Version 10.5 software measured the left side of the footprints, i.e. the normal side (N), and the right side, i.e. the surgical side (E), the parameters: the furthest distance between the heel and the tip of the foot (print length (PL)), with EPL representing the length of the footprint on the surgical side and NPL representing the length of the footprint on the normal side; the distance of the opening from the first toe to the fifth toe (toe spread (TS)), with ETS representing the width of the toe spread on the surgical side and NTS representing the width of the toe spread on the normal side; and the width of the intermediate toe spread from the second toe to the fourth toe (intermediary toe spread (ITS)), with EITS being the width of the intermediate toe on the operative side and NITS being the width of the intermediate toe on the normal side (Figure 7A). Sciatic functional index (SFI) was calculated according to the Bain SFI score [25]:

(2) SFI=−38.3(EPL−NPL)/NPL+109.5(ETS−NTS)/NTS+13.3(EITS−NITS)/NITS−8.8

The given formula was used to calculate the value of SFI. The normal sciatic nerve function is indicated by SFI = 0, while full loss of sciatic nerve function is SFI = −100.

#### Electrophysiological evaluation

A multichannel electrophysiologic signal acquisition system was used to obtain experimental electrophysiologic images to evaluate postoperative sciatic nerve recovery in rats. Compound muscle action potentials (CMAP) were detected by electrophysiological instruments. Bipolar stimulating electrodes from a multichannel electrophysiology system were used to stimulate the proximal and distal nerve trunks of the regenerated sciatic nerve after the proximal and distal ends of the anastomosis were exposed. The electrodes were then inserted into the anterior tibial muscle. Then, the nerve was stimulated with continuously intensified electrical pulses until a response was produced, then the distance between the distal and proximal positions of the stimulating electrodes and the receiving electrodes, the latency and the CMAP were recorded, and the motor nerve conduction velocity (MCV) was calculated to assess the recovery of the sciatic nerve of the rats after surgery.

#### Toluidine blue staining of regenerating nerve

The distal nerve sections next to the anastomosis location were removed after electrophysiological testing (Figure 7E). Axonal regeneration and myelin growth were assessed for each group through toluidine blue staining of the nerves The specific steps were as follows: (i) After removing the distal nerve, it was placed in 2.5% (v/v) glutaraldehyde and soaked for ∼2 h at 4°C. Then fixed for roughly 30 min using 1% (v/v) osmium tetroxide; (ii) after gradient alcohol dehydration, the embedding was carried out by Epon 812 epoxy resin, and the embedded samples were semi-thinly sliced by ultra-thin slicer, with the thickness of 0.5 μm; (iii) section staining was performed using a 1% toluidine blue staining solution; (iv) the results were observed with light microscope and images were captured; and (v) the myelinated axons’ density (measured as the number of axons/mm^2^) was quantified utilizing ImageJ software.

#### Detection of specific markers for regenerating nerves

The connective tissue of the distal nerve was removed, immersed in paraformaldehyde solution, and fixed for ∼12 h. Following a series of 20% and 30% sucrose solutions dehydration steps, OCT（optimal cutting temperature compound）-embedded frozen sections measuring 8 μm in thickness were created. Fluorescence staining was performed. In short, a mixed antibody dilution of Tuj1 (dilution ratio of 1:100) and S-100 (dilution ratio of 1:200) was added dropwise to the slices, then which were kept at 4°C for the whole night. The fluorescently labeled secondary antibody dilution (dilution ratio 1:500) was dropped and incubated at 37°C for 1 h, and finally blocked with an anti-fluorescence quencher. The staining results were observed by confocal microscopy, the graphs were captured, and for analysis, the top, bottom, left, right, and center fields of vision were selected. The Tuj1 positive ratio of the area was determined by ImageJ software.

### Statistical analyses

SPSS 25.0 statistical software was used to handle and analyze the data. The data obtained were expressed as mean ± standard deviation. Comparisons between two groups were analyzed by *t*-test, and comparisons between multiple groups were analyzed by two-way analysis of variance. ns indicates that the difference is not statistically significant, and *P *<* *0.05 means that the difference is statistically significant.

## Results

### Decellularization efficiency analysis

**Figure 1.**
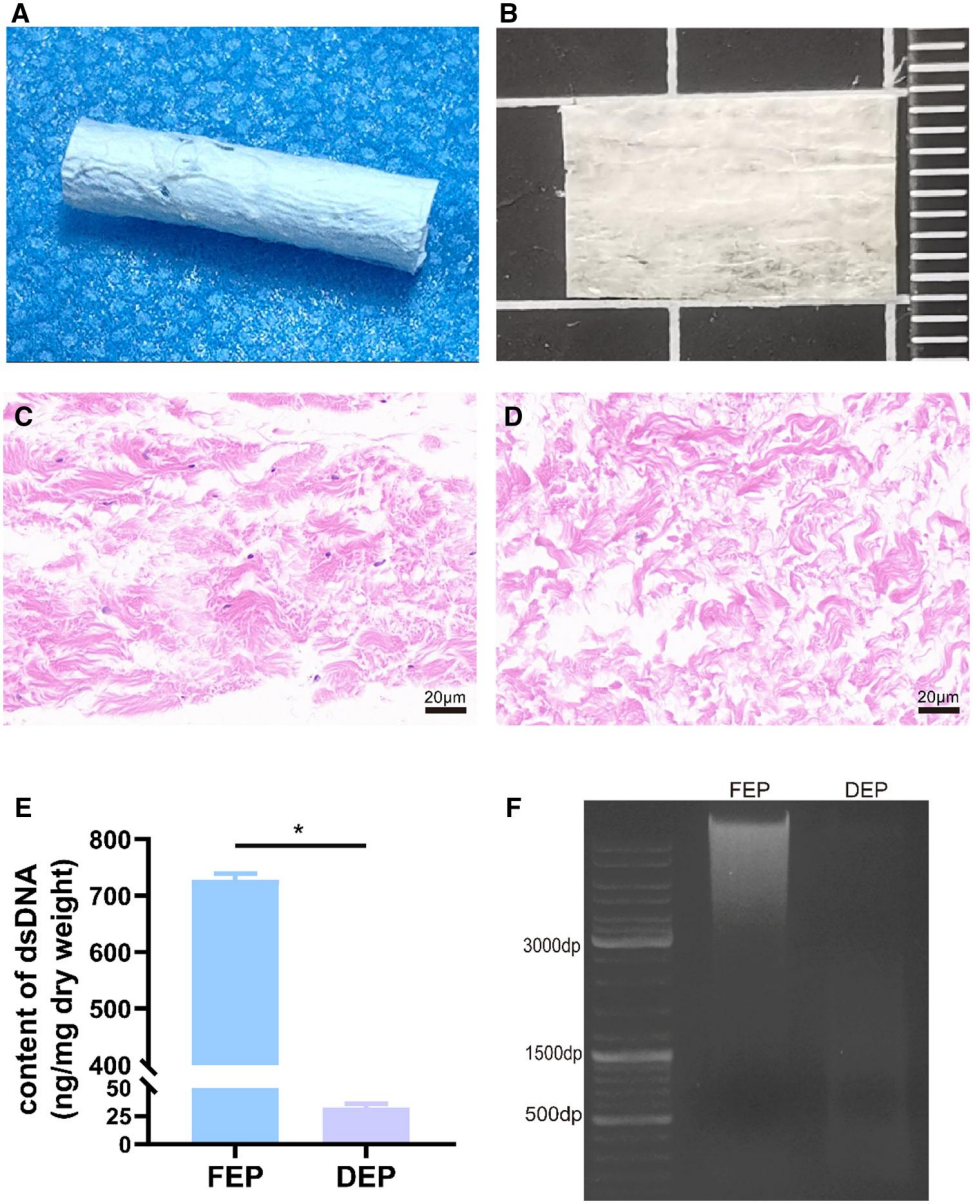
Assessing the epineurium’s decellularization effectiveness. FEP: Fresh epineurium; DEP: decellularized epineurium. Decellularized epineurium (**A** and **B**). H&E staining of fresh epineurium (**C**). H&E staining of decellularized epineurium (**D**). Quantitative analysis of dsDNA (**E**). DNA agarose gel electrophoresis (**F**). Data are expressed as the mean ± SD (*n* = 5). *P <* *0.05.

The effectiveness of decellularization was evaluated using tissue staining and biochemical analysis. When compared to FEP, DEP had lower number of nuclei, as demonstrated by H&E staining. In DEP, agarose gel electrophoresis did not reveal any discernible DNA bands. In addition, analysis of residual DNA content showed a significant reduction in DEP compared with FEP (*P *<* *0.05), with DEP’s DNA level falling short of the 50 ng/mg international norm (Figure 1). These findings validate the effectiveness of decellularization by showing that it satisfies the established standards [26].

### GAGs content in DEP and FEP

GAGs are essential for nerve regeneration. In FEP and DEP, the GAGs content was 9.46 ± 0.36 and 3.39 ± 0.48 μg/mg of dry tissue, respectively, which revealed a notable reduction in the DEP group compared to the FEP group (*P* < 0.05) (Supplementary Figure S1).

### Proteomics analysis

#### Coexisting proteins in FEP and DEP

**Figure 2.**
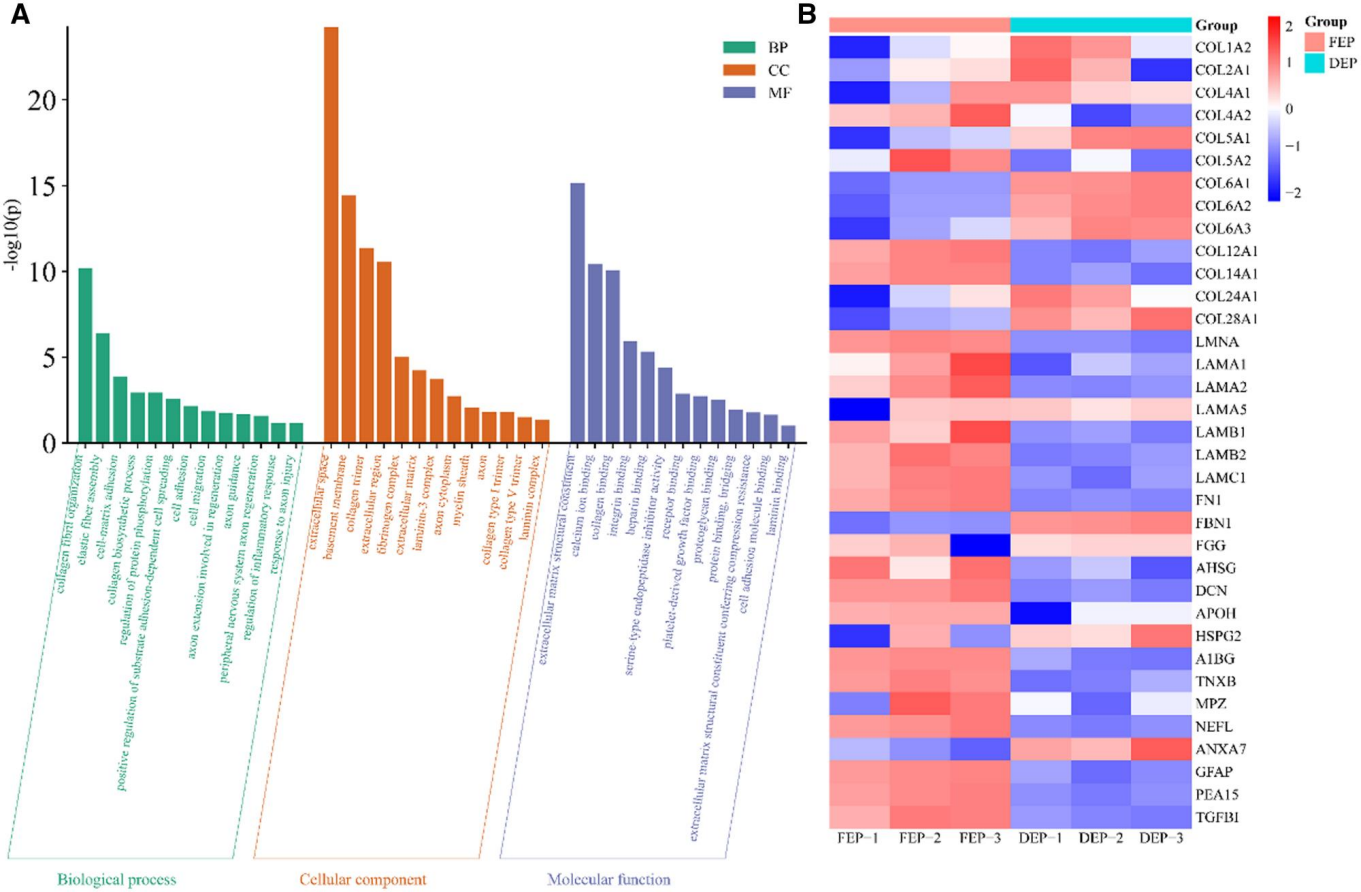
Coexisting protein analysis. Protein gene ontology enrichment analysis (**A**). The analysis of protein abundance for certain identified proteins. The bluer color, the lower the in relation content of protein (**B**). AHSG, α2-HS glycoprotein; AIBG, α1-beta glycoprotein; ANXA7, annexin; APOH, beta 2 glycoprotein I; BP, biological process; CC, cellular components; COL1A2, collagen type I alpha 2 chain; COL2A1, collagen type II alpha 1 chain; COL4A1, collagen type IV alpha 1 chain; COL4A2, collagen type IV alpha 2 chain; COL5A1, collagen type V alpha 1 chain; COL5A2, collagen type V alpha 2 chain; COL6A1, collagen type VI alpha 1 chain; COL6A2, collagen type VI alpha 2 chain; COL6A3, collagen type VI alpha 3 chain; COL12A1, collagen type XII alpha 1 chain; COL14A1, collagen type XIV alpha 1 chain; COL24A1, collagen type XIV alpha 1 chain; COL28A1, collagen type XXVII alpha 1 chain; DEP, decellularized epineurium; DCN, decorin; FBN1, fibrillin-1; FN1, fibronectin; FGG, fibrinogen γ; FEP, fresh epineurium; GFAP, glial fibrillary acidic protein; HSPG2, heparan sulfate proteoglycan 2; LAMA1, laminin subunit alpha 1; LAMA2, laminin subunit alpha 2; LAMA5, laminin subunit alpha 5; LAMB1, laminin subunit beta 1; LAMB2, laminin subunit beta 2; LAMC1, laminin subunit gamma 1; LMNA, lamin filament; MF, molecular functions; MPZ, myelin protein; NEFL, neurofilament; PEA15, phosphoprotein enriched in astrocytes 15; TGFB1, transforming growth factor, beta 1; TNXB, tenascin.

The GO analysis of proteins commonly present in both FEP and DEP reveals that the majority of these proteins are concentrated in the ECM components (Figure 2A). Specifically, from the perspective of biological processes, they predominantly participate in the organization of collagen fibrils and promote the migration and adherence of cells. The relevant terms include collagen fibril organization (GO: 0030199), elastic fiber assembly (GO: 0048251), cell-matrix adhesion (GO: 0007160), collagen biosynthetic process (GO: 0032964), cell adhesion (GO: 0007155), cell migration (GO: 0016477) and axon extension involved in regeneration (GO: 0048677). In terms of cellular components, they are primarily associated with the ECM, such as collagen and basement membranes, with relevant terms including extracellular space (GO: 0005615), basement membrane (GO: 0005604), collagen trimer (GO: 0005581), extracellular region (GO: 0005576), ECM (GO: 0031012) and collagen type I trimer (GO: 0005584). Furthermore, from a molecular function perspective, most are related to protein binding, as indicated by terms such as ECM structural constituent (GO: 0005201), collagen binding (GO: 0005518), protein binding, bridging (GO: 0030674), cell adhesion molecule binding (GO: 0050839) and LN-binding (GO: 0043236).

Enrichment analysis was performed on the protein constituents detected in both FEP and DEP samples (Figure 2B). The analysis revealed a substantial presence of collagen and basement membrane proteins, such as types I, II, IV V and VI collagens (COL I, COL II, COL IV, COL V, COL VI), along with LNs (LAMA, LAMB) and FN, in the epineurium both before and after decellularization. Additionally, there was an observed increase in the relative abundance of certain collagen proteins. The epineurium also contained a variety of glycoproteins, including but not limited to alpha-2-HS-glycoprotein, decorin, heparan sulfate proteoglycan 2, and alpha-1β-glycoprotein. Furthermore, a minor complement of cytokines, such as transforming growth factor beta 1 (TGF-β1), was also present in the epineurium.

#### Comparisons of distinct proteins in FEP and DEP

Differential analysis was conducted on the identified proteins, with differential proteins (DEPs) being selected based on a threshold of *P* < 0.05 and an absolute log2 fold change (|log2FoldChange|) of at least 1. This analysis resulted in the identification of 116 DEPs, consisting of 100 proteins with decreased relative abundance and 16 proteins with increased relative abundance. The GO analysis was utilized to elucidate the biological function disparities among these DEPs.

**Figure 3.**
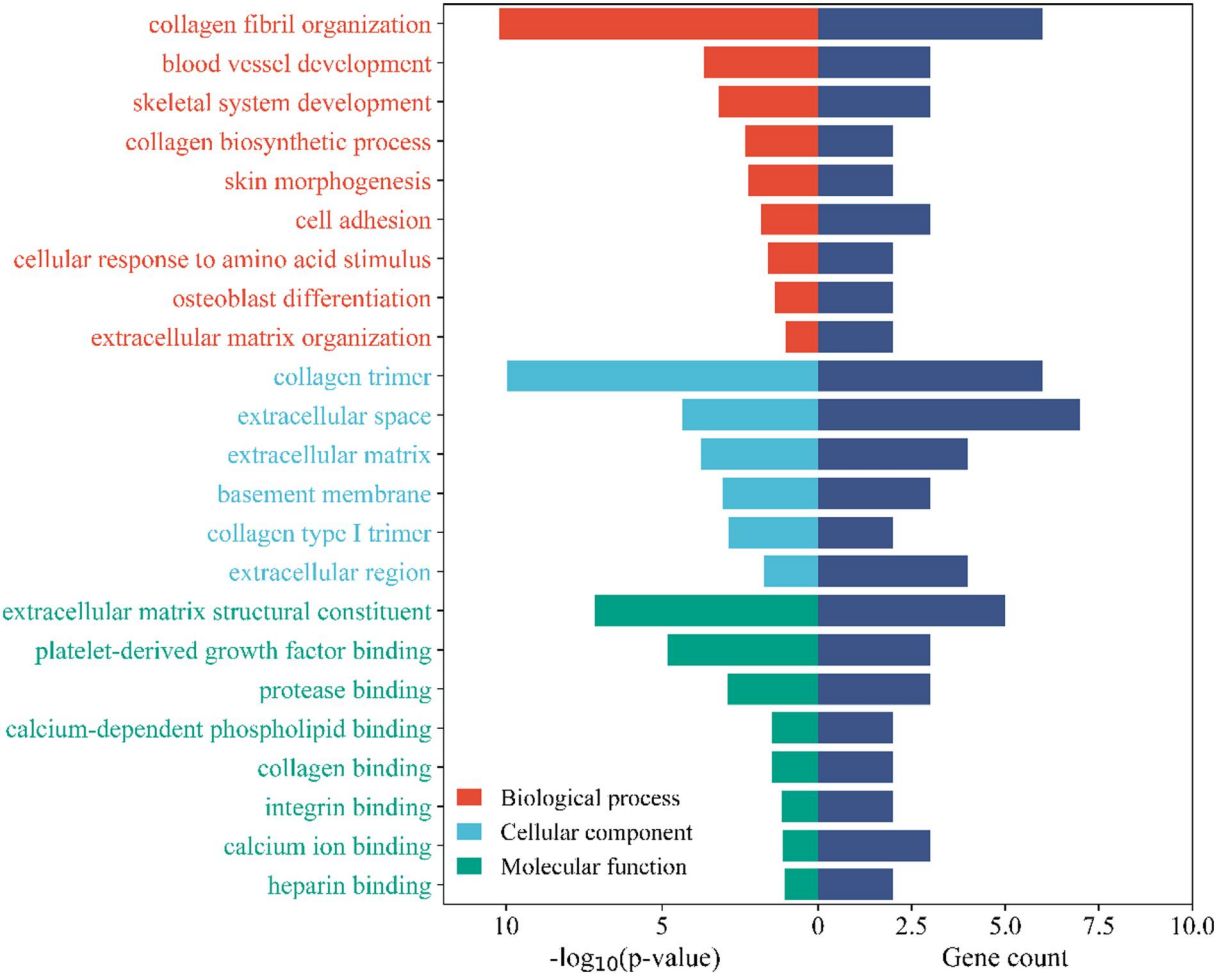
GO analysis of up-regulated proteins. The horizontal bars are categorized by color to indicate three distinct GO terms: Biological process (red), cellular component (blue) and molecular function (green). The *x*-axis shows two scales: on the left, there is the negative logarithm to the base 10 of the *P* values (−log10(*P* values)), which indicates the statistical significance of each term; on the right, the gene count, which denotes the number of genes associated with each term.

The GO analysis's findings indicate that the proteins with increased relative abundance primarily play a crucial part in the structure and function of the ECM (Figure 3). A significant number of these genes contribute to collagen-related structures and functions and their interactions with other molecules. For the category of Biological Processes, the relevant terms include collagen fibril organization (GO: 0030199), blood vessel development (GO: 0001568), collagen biosynthetic process (GO: 0032964) and cell adhesion (GO: 0007155). Regarding the Cellular Component category, implicated terms are collagen trimer (GO: 0005581), extracellular space (GO: 0005615), ECM (GO: 0031012) and basement membrane (GO: 0005604). As for Molecular Function, some of the associated terms are platelet-derived growth factor binding (GO: 0048407), ECM structural constituent (GO: 0005201), protease binding (GO: 0002020) and collagen binding (GO: 0005518).

**Figure 4.**
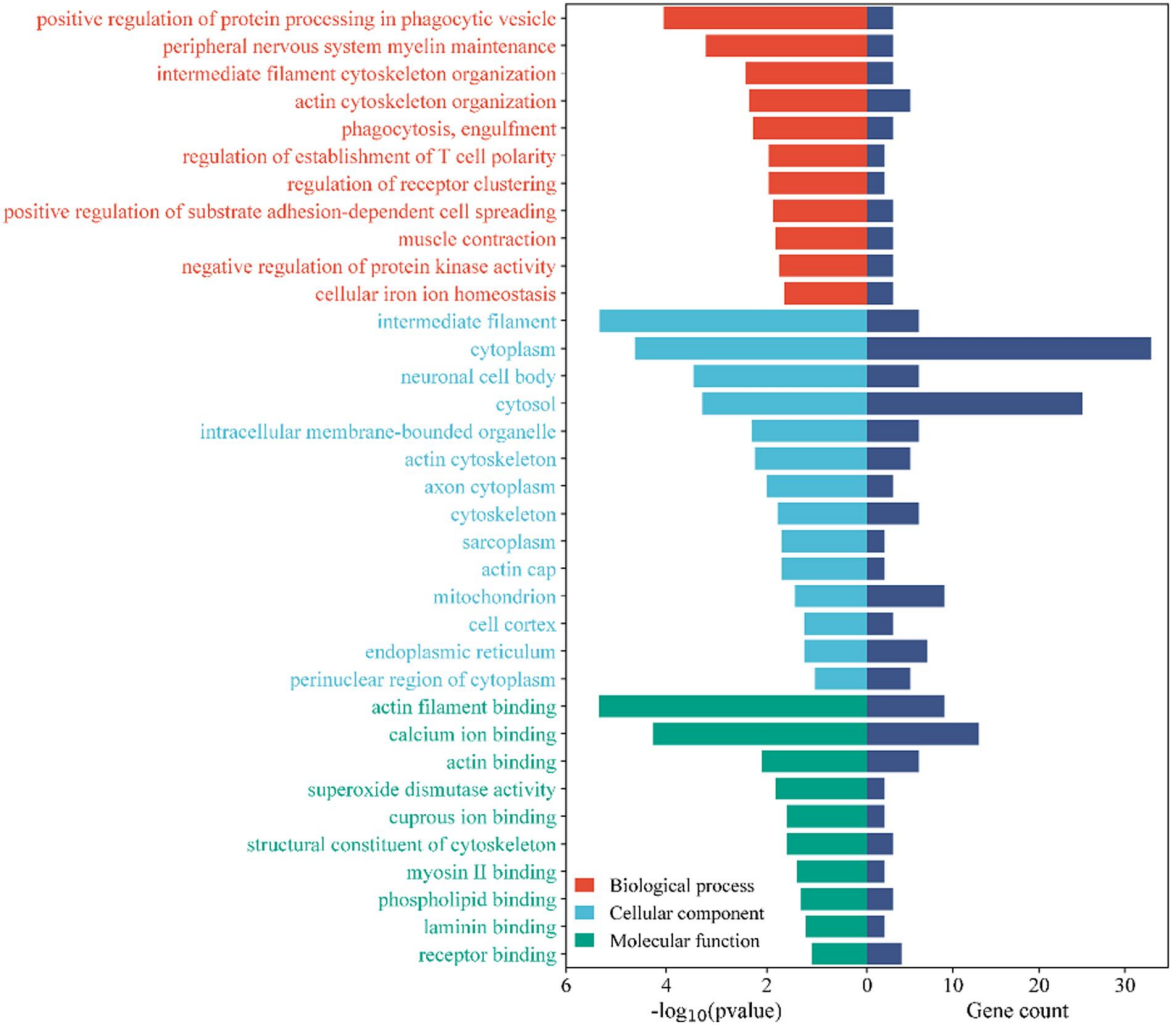
GO analysis of down-regulated proteins. The horizontal bars are categorized by color to indicate three distinct GO terms: biological process (red), cellular component (blue) and molecular function (green). The *x*-axis shows two scales: on the left, there is the negative logarithm to the base 10 of the *P* values (−log10(*P* values)), which indicates the statistical significance of each term; on the right, the gene count, which denotes the number of genes associated with each term.

The proteins exhibiting decreased relative abundance are predominantly associated with cellular structure and activity (Figure 4). Within the Biological Process category, the processes implicated include intermediate filament cytoskeleton organization (GO: 0045104), positive regulation of protein processing in phagocytic vesicle (GO: 1903923), actin cytoskeleton organization (GO: 0030036) and regulation of establishment of T cell polarity (GO: 1903903). In terms of Cellular Components, the relevant terms are intermediate filament (GO: 0005882), cytoplasm (GO: 0005737), cytosol (GO: 0005829), and intracellular membrane-bounded organelle (GO: 0043231). For Molecular Function, some pertinent terms include calcium ion binding (GO: 0005509), actin filament binding (GO: 0051015), actin binding (GO: 0003779) and superoxide dismutase activity (GO: 0004784).

### Morphology and structure of the DEP

The microstructure of the DEP was observed using via SEM, and it was noted that the surface of DEP is relatively flat with no obvious pores. (Supplementary Figure S2).

### Water absorption

In order to promote the interchange of nutrients and metabolites and support the nerve regeneration, effective water absorption is essential. In this study, the water absorption rate of FEP was 5.729 ± 0.319, while the decellularization epineurium was 7.771 ± 1.528, indicating that decellularization treatment could increase the water absorption of epineurium.

### Cytotoxicity

**Figure 5.**
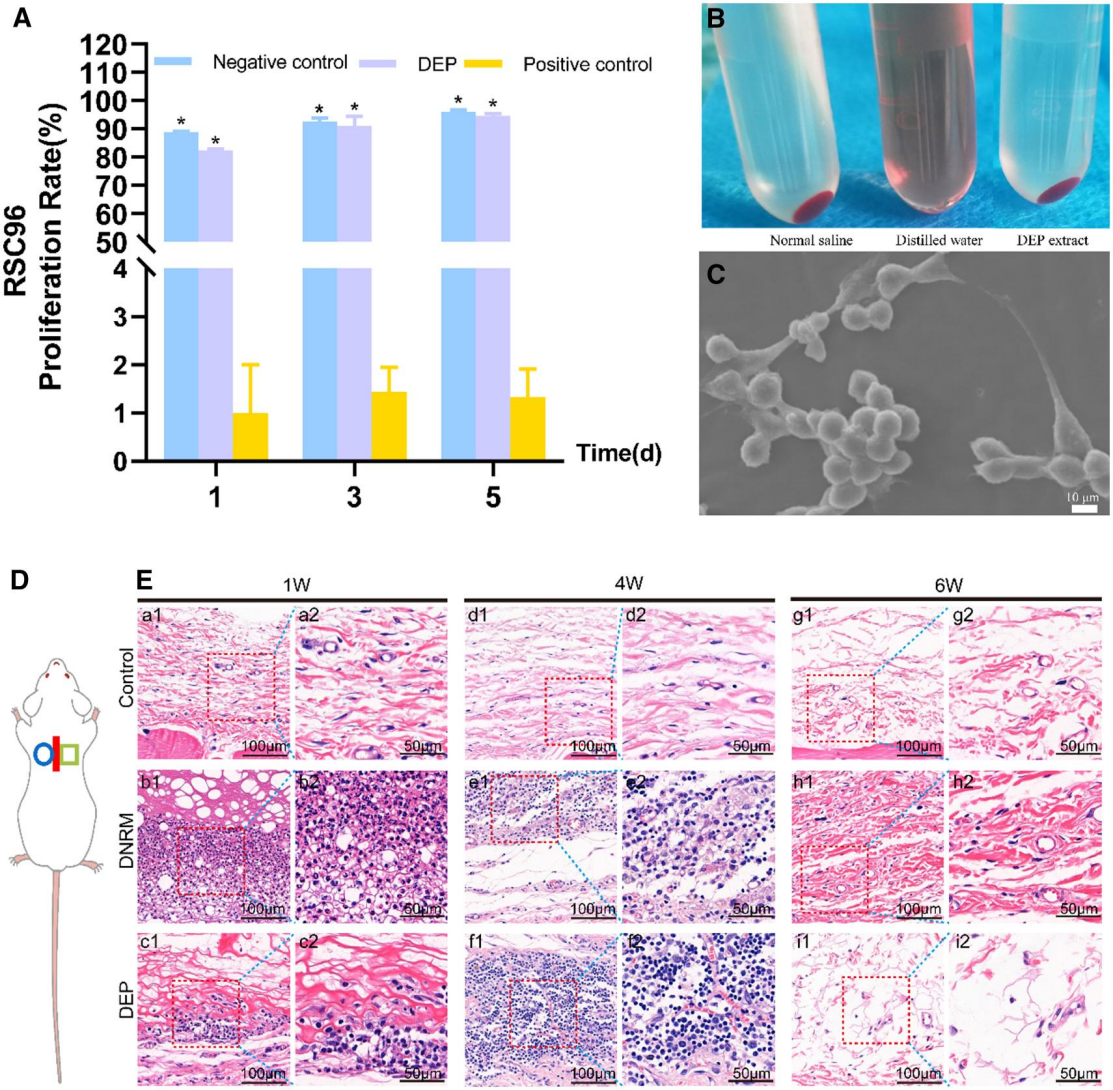
Characterization and biocompatibility of DEP. CCK-8 test of the extract medium was used to evaluate the cytotoxicity of DEP (**A**). The test’s qualitative hemolysis results (**B**). The negative control of normal saline exhibited no hemolysis, and the DEP extract showed no signs of causing hemolysis. In contrast, the positive control of distilled water demonstrated hemolysis. The adherence of Schwann cells on the surface of DEP was examined via SEM at ×1000 magnifications on the third day (**C**). Schematic diagram of experimental subcutaneous implantation on the back of rats (**D**), the circle on the left represents the control group, and the box on the right represents the material group. H&E staining at various times (**E**). Control, no material. (a1, b1, c1, d1, e1, f1, g1, h1, i1, scale bar = 100 μm). The dotted red box is observed at a higher magnification (a2, b2, c2, d2, e2, f2, g2, h2, i2, scale bar = 50 μm). Data are expressed as the mean ± SD (*n* = 5). *P <* *0.05, compared with positive control group.

The CCK-8 assay is a colorimetric method that evaluates metabolic activity of cells as an indicator for cytotoxicity and cell growth (Figure 5A). The results showed that at all-time points (1, 3 and 5 days), the negative control group exhibits a constant increase in proliferation of cells. The DEP group also shows an increase in proliferation of cells, similar to the negative control, which suggests that the DEP does not exhibit cytotoxic effects on the cells. On the other hand, the positive control group displays a significantly decreased cell proliferation rate. In summary, the tested material (DEP) does not appear to have a cytotoxic effect on the cell culture within the observed time frame of the experiment, as the proliferation rates are comparable to those of the negative control and significantly higher than the positive control (*P *<* *0.05). This suggests that DEP is potentially biocompatible and safe for the cells.

### Hemolysis test

The hemocompatibility of the DEP was assessed via a hemolysis assay. In the negative control, physiological saline was used, resulting in erythrocyte sedimentation with a clear and colorless supernatant, indicating no hemolysis. Conversely, distilled water served as the positive control, leaving no residual erythrocytes at the bottom of the tube, indicative of complete hemolysis. DEP extract supernatant retained its clear, colorless look, exhibiting no significant change from the negative control. It suggests that contact with the tubular material did not induce hemolysis (Figure 5B). The HR quantifies the extent of erythrocyte rupture and dissolution resulting from material contact. In standardized hemolysis assessments, substances with an HR below 2% are categorized as non-hemolytic, while those with HR between 2% and 5%, or higher, is classified as little hemolytic or hemolytic, correspondingly. As Table 1 illustrates, the HR for DEP was 1.11%, classifying it as a non-hemolytic material, demonstrating favorable hemocompatibility.

**Table 1.** OD 545 nm value and HR in three groups based on the hemolysis assay

Group	OD_545_	HR (%)
DEP extract	0.0504 ± 0.0132	1.11
Distilled water	0.259 ± 0.002	100
Normal saline	0.0475 ± 0.0005	0

### Cell affinity assay

In order to evaluate the affinity between DEP and Schwann cells, SEM was employed to analyze cell morphology and adhesion in cultivated together cells and materials (Figure 5C). From the image, we can observe that the Schwann cells have attached to and spread across the surface of the material, the three-dimensional sense is strong, and the elongated and slender protrusions are interwoven into a network. This indicates that the DEP has a good affinity for cells and can promote cell growth and adhesion, which is an indicator of good biocompatibility.

Following three days of Schwann cell culture in DEP extract, confocal microscopy images showed stretched morphology of Schwann cells (Supplementary Figure S3).

### H&E Staining of subcutaneous implants in rats

In the subcutaneous implantation experiments, the immunological inflammatory response induced by the material after implantation was analyzed through H&E staining and immunohistochemical staining. Without any issues, every rat lived until the scheduled time intervals. H&E staining indicated that from the first to the fourth week, there was significant inflammatory cell infiltration in both the DNRM group and the DEP group in contrast to the control group. However, by the sixth week, the infiltration of inflammatory cells was markedly reduced (Figure 5E).

### Immunohistochemical observation of subcutaneous implants in rats

**Figure 6.**
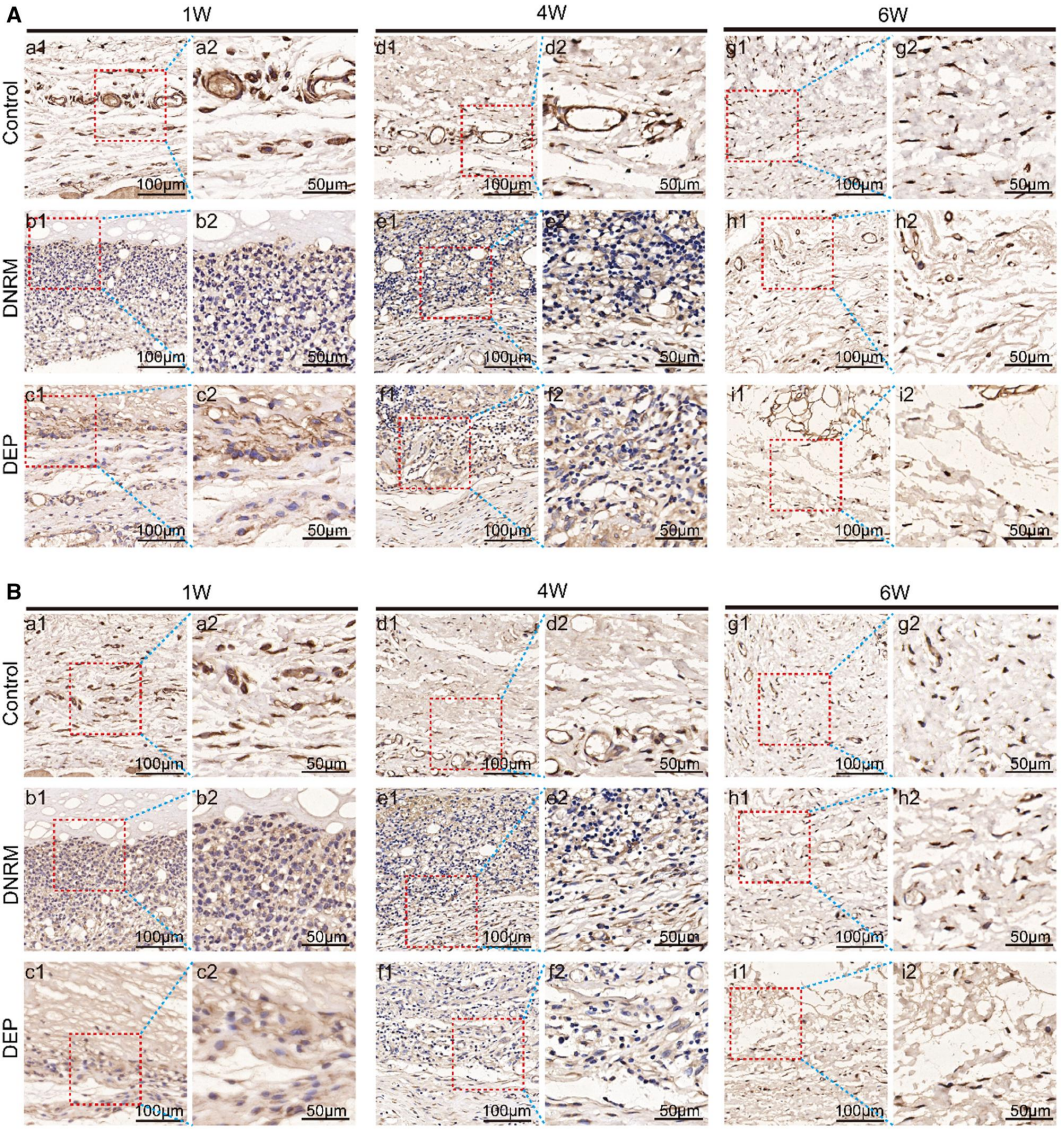
Samples stained with immunohistochemicals at different times, where CD11b signifies neutrophils (**A**) and CD68 indicates macrophages (**B**). Control, no material. (a1, b1, c1, d1, e1, f1, g1, h1, i1, scale bar = 100 μm). The red box with dots is seen at a higher magnification (a2, b2, c2, d2, e2, f2, g2, h2, i2, scale bar = 50 μm).

Figure 6 presents the results of immunohistochemical staining for CD11b and CD68 on the dorsal subcutaneous implants from rats. CD68 is used to label macrophages, while CD11b marks neutrophils. Positive staining is indicated by cells that exhibit brownish or light brown cytoplasm.

The staining results for neutrophils labeled with CD11b indicate that there was no significant inflammatory response from the first to the sixth week in the control group. From the results of the DNRM group, infiltration of neutrophils began in the first week, but there was no cellular invasion into the membrane. The DEP group, however, showed a small amount of cell infiltration, with some positive expression found within the membrane. By the fourth week, both material groups exhibited a large amount of positive expression and began to progressively infiltrate into the interior of the materials. By the sixth week, there was a notable decrease in CD11b expression (Figure 6A). In addition, according to the CD11b positive area ratio, the positive expression of neutrophils was the highest in the first week, and the DNRM group had the most severe infiltration (*P *<* *0.05). The positive expression rate of each group gradually decreased over time, and by the sixth week, the three groups’ neutrophil positive area ratios did not significantly differ from one another (*P *>* *0.05) (Supplementary Figure S4A).

The immunohistochemical results for the macrophage marker CD68 showed that, compared to the control group (control), both material groups exhibited varying degrees of CD68 positive expression, which gradually diminished by the sixth week. Among them, the positive expression rate of the DEP was slightly lower than that of the DNRM (Figure 6B). According to the CD68 positive area ratio, in contrast to the control group, the DNRM group had the highest positive expression rate of macrophages (*P *<* *0.05) until the sixth week, which was not significantly distinct with the control group, while the DEP group was closer to the control group (*P *>* *0.05) (Supplementary Figure S4B).

### *In vivo* functional study

#### Claw-spread reflex analysis

Assessment of the claw-spread reflex provides insights into the recovery status of nerve function post-sciatic nerve injury, graded from Grade A (normal function) to Grade C (complete functional impairment) (Table 2). At 4 weeks post-neurorrhaphy, the majority of rats in the DEP group were graded as B (responsive to needle prick but without toe extension reflex), with a minority assessed at Grade C (no response to needle prick); the DNRM group displayed an equal distribution of rats at grades B and C; while the majority of the control group were at Grade C. Eight weeks after the procedure, 50% of rats in the DEP group were at Grade A (responsive to needle prick with toe extension reflex) and 50% at Grade B; in the DNRM group, the majority were Grade B, accounting for 66.67%, with grades A and C each representing 16.67%; in the control group, Grade B rats were the most prevalent at 50%, while Grade A was the least common, at 16.67%.

**Table 2.** Number and proportion of rats in each group determined by claw-spread reflex test

Group	Grade A, *n* (%)	Grade B, *n* (%)	Grade C, *n* (%)
4 W			
DEP	0	4 (66.67%)	2 (33.33%)
DNRM	0	3 (50%)	3 (50%)
Control	0	2 (33.33%)	4 (66.67%)
8 W			
DEP	3 (50%)	3 (50%)	0
DNRM	1 (16.67%)	4 (66.67%)	1 (16.67%)
Control	1 (16.67%)	3 (50%)	2 (33.33%)

#### Assessment of tissue adhesion at the nerve anastomosis site

Adhesions at the neurorrhaphy site were assessed at the fourth and eighth weeks postoperatively. In the week, extensive degradation was observed in DNRM, while DEP exhibited only partial degradation. By the eighth week post-surgery, DEP had completely degraded. As shown in Table 3, in the fourth week, the number of rats graded level 1, wrapped with DNRM and DEP, was higher than that in the control group. In the eighth week, both groups had more rats with a score of 0 in contrast to the control group. The DEP group had higher scores at both points compared to the DNRM group, demonstrating DEP's superior anti-adhesion effects.

**Table 3.** Number of rats at each grade according to the adhesion assessment at anastomosis sites

Group	Grade 0	Grade 1	Grade 2
4 W			
DEP	0	4	2
DNRM	0	3	3
Control	0	1	5
8 W			
DEP	3	3	0
DNRM	1	5	0
Control	0	3	3

#### SFI evaluation

**Figure 7.**
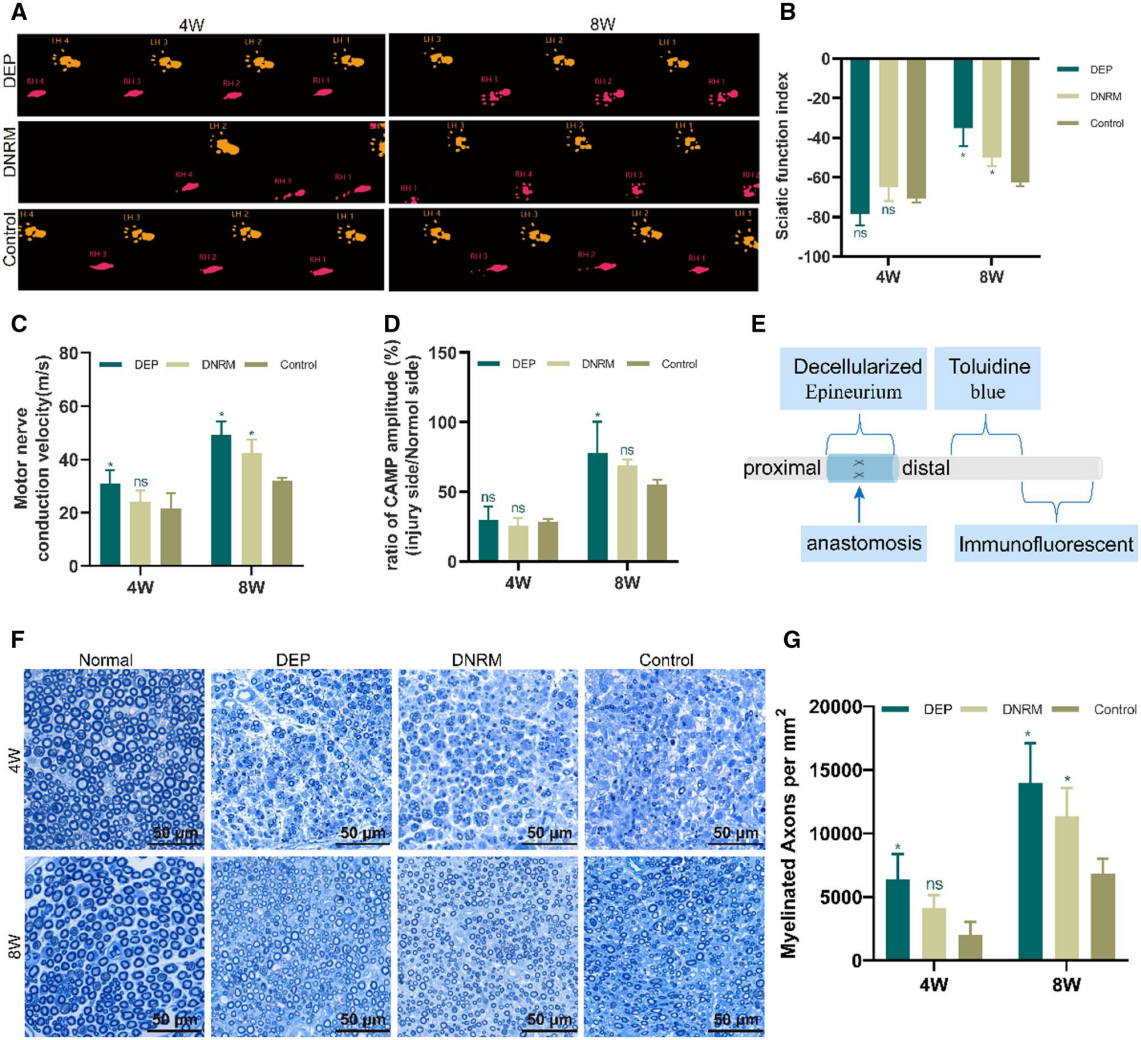
Assessment of the *in vivo* nerve regeneration’s reparative impact in rats. Postoperative weeks 4 and 8 footprint patterns of rats in each group (**A**). SFI values of each group at 4 and 8 weeks post-surgery (**B**). MCV (**C**) and compound action muscle potential (CAMP) amplitude ratio (**D**) for each group at 4 and 8 weeks post-surgery. Schematic diagram of postoperative tissue sampling and staining (**E**). Analysis of toluidine blue staining results for regenerated myelinated nerves at 4 and 8 weeks post-surgery (**F**). Analysis results of myelinated nerve fiber density (**G**). Data are represented as mean ± standard deviation (*n* = 6), with comparisons between multiple groups made using two-way analysis of variance. *P <* *0.05, compared with the control group. Control, without materials wrapping; LH, left hind healthy side; RH, right hind operative side.

SFI in the DEP, DNRM, and control groups all showed a trend of increase over time. In the fourth week post-surgery, there was no significant difference in the SFI between the DEP and DNRM groups in contrast to the control group (*P *>* *0.05). However, at the eighth week post-surgery, the SFI of the DEP and DNRM groups was higher than that of the control group, exhibiting a difference that is statistically significant (*P *<* *0.05) (Figure 7B).

#### Electrophysiological analysis

At the fourth and eighth weeks post-surgery, through electrophysiological experiments, the regenerated nerves’ functional recovery was evaluated. After surgery, about the fourth week, the MCV in the DEP group was significantly higher than in the control group (*P *<* *0.05), but there was no significant difference in the CMAP compared to the control group (*P *>* *0.05). The MCV and CMAP amplitude ratio in the DNRM group did not show a significant difference in contrast to the control group (*P *>* *0.05). At the eighth week post-surgery, both the MCV and CMAP amplitude ratio in the DEP group were significantly higher than those in the control group (*P *<* *0.05). The MCV in the DNRM group was also significantly higher than in the control group (*P *<* *0.05), but the compound action muscle potential showed no difference in contrast to the control group (*P *>* *0.05) (Figure 7C and 7D).

#### Analysis of toluidine blue staining

The density of myelinated fibers in regenerated nerves was evaluated using toluidine blue staining. It was observed that at the fourth week post-surgery, the density and number of myelinated sheaths in the DEP, DNRM, and control groups were significantly reduced compared to normal nerve myelin (Figure 7F). Among these, the number of regenerated myelin sheaths in the DEP group was significantly higher compared to the control group (*P *<* *0.05), while the number in the DNRM group did not show a significant difference compared to the control group (*P *>* *0.05). At the eighth week post-surgery, the number of myelin sheaths in all three groups was significantly higher than at the fourth week, with thicker myelin diameters. Compared to the control group, both the DEP and DNRM groups revealed a difference in the number of regenerated myelin sheaths that was statistically significant (*P *<* *0.05) (Figure 7G).

#### Immunofluorescence analysis of regenerating nerve fibers

At the fourth and eighth weeks after transplantation, slices of the renewed nerve were subjected to immunofluorescence staining with two distinct markers, S-100 and Tuj1, to observe nerve regeneration. S-100, a protein that nourishes neurons, is typically secreted by Schwann cells in the peripheral nervous system. Hence, immunofluorescence staining for the S-100 marker could observe the proliferation and migration of Schwann cells throughout the whole process of nerve repairment and regeneration. Tuj1 is a microtubule protein considered to be involved in neuron-specific differentiation and can serve as an early neuronal characteristic immunomarker. In the confocal microscope images, green represents S-100 protein, red indicates Tuj1 protein and blue is for cell nuclei.

**Figure 8.**
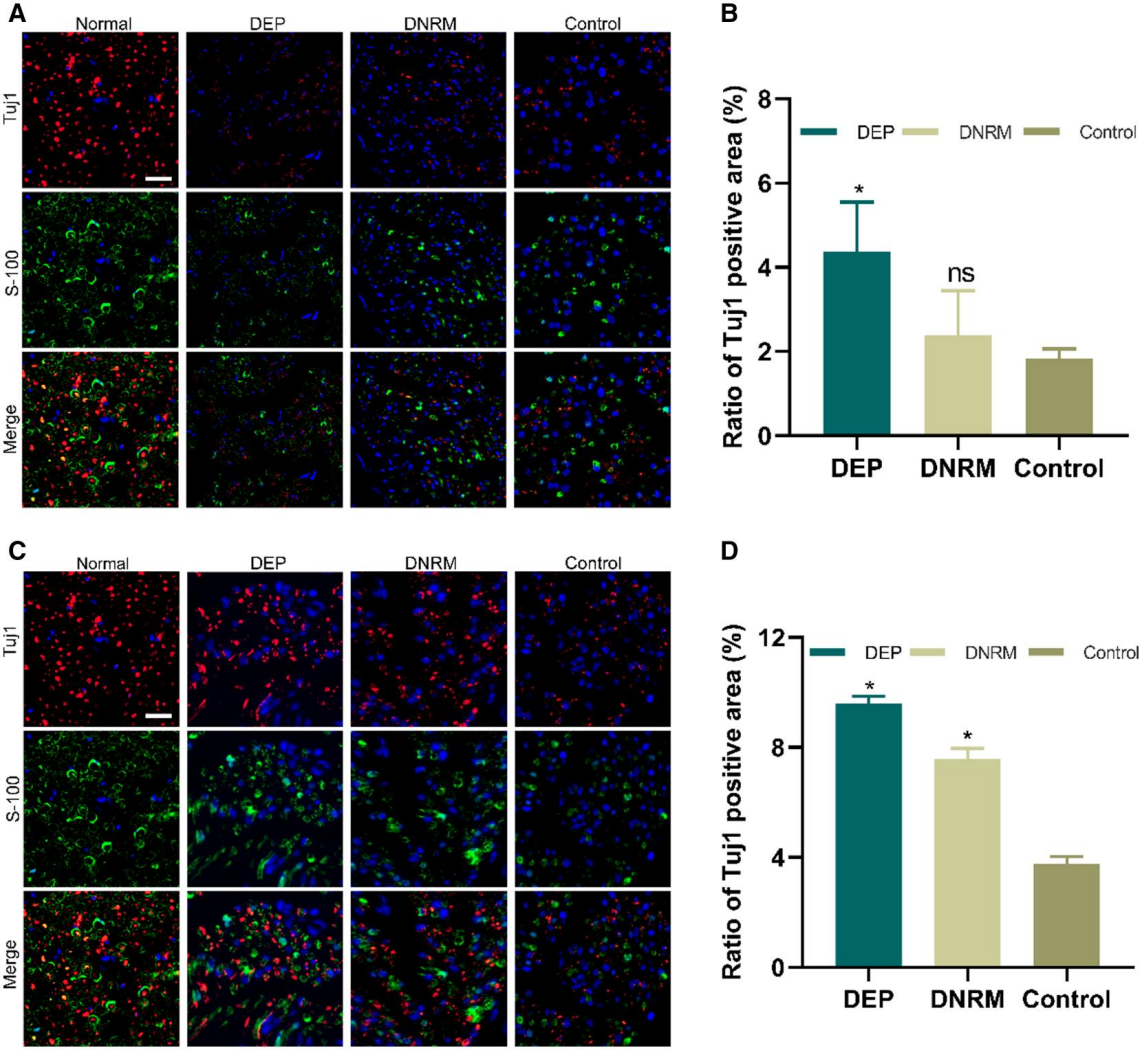
Immunofluorescence staining results of Tuj1 and S-100 in nerve samples. Immunofluorescence staining results of regenerated nerves at the fourth week post-surgery (**A**) and the positive area ratio of Tuj1 in regenerated nerves (**B**). Immunofluorescence staining results of regenerated nerves at the eighth week post-surgery (**C**) and the positive area ratio of Tuj1 in regenerated nerves (**D**). Scale = 20 μm. Data are represented as mean ± standard deviation (*n* = 6), with comparisons between multiple groups made using two-way analysis of variance. *P <* *0.05, compared with the control group. Control, without materials wrapping group; Normal, normal nerve.

From Figure 8A, it can be observed that in the fourth week after surgery, in normal nerves, the intensity of both red and green fluorescence is higher compared to the other surgical groups. The green and red fluorescence intensities of the three surgical groups are not significantly different, indicating that no obvious nerve regeneration had occurred by the fourth week.

At the eighth week post-surgery, the intensity of both green and red fluorescence in all three groups was significantly stronger than at the fourth week. Among these, the fluorescence intensity in the DEP and DNRM groups was noticeably stronger than in the control group, with the regenerated myelin sheaths in the DEP group being closest to that of normal nerves (Figure 8C).

Through immunofluorescence staining, the positive area ratio of Tuj1 was calculated to assess the quality of the regenerated nerve (Figure 8B and D). From the fourth to eighth week post-surgery, the positive area ratio of Tuj1 gradually increased. In the fourth week post-surgery, compared with the control group, DEP showed a significant increase in the positive area ratio of Tuj1 fluorescence (*P *<* *0.05), while there was no significant difference between the DNRM and the control group (*P *>* *0.05) (Figure 8B). At the eighth week post-surgery, both the DEP and DNRM groups exhibited a significantly higher positive area ratio of Tuj1 fluorescence compared with the control group (*P *<* *0.05) (Figure 8D).

## Discussions

The conventional method for repairing severed peripheral nerves often entails directly suturing the nerve ends. Nonetheless, this approach may result in adhesion of the nerve anastomosis to adjacent tissues or the development of neuromas, impeding the restoration of nerve function [3, 27]. Thus, we have devised an innovative xenogeneic DEP as an anti-adhesion membrane, aimed at averting adhesions and fostering nerve repairment and regeneration.

This study utilized the epineurium of the sciatic nerve derived from pigs, which was decellularized and then used as an anti-adhesion membrane at the site of nerve anastomosis. Compared to complete nerve tissue, the epineurium is thinner and contains fewer cells. Therefore, this study improved the Sondell method for decellularization. Based on the Sondell method [28], we performed only a single round of decellularization. The results showed that after the decellularization process, H&E staining did not reveal any visible cell nuclei, and the DNA content measured by quantitative DNA detection was significantly less than the standard of 50 ng/mg. In addition, the DEP did not exhibit any discernible DNA bands in the DNA gel electrophoresis results. Therefore, that could be shown that the modified Sondell decellularization technique employed in this work produced the DEP in an effective way, eliminating the cellular components and satisfying the criteria put forth by Crapo [26] and other scholars.

By modifying the activity of the corresponding tissue cells, the physiologically active chemicals found in the ECM are essential for tissue regeneration [29–31]. This study analyzed the compositional structure of the epineurium both prior to and following decellularization using proteomics. The findings indicated that the epineurium contains an abundance of ECM components, like collagen, FN, LN, and proteoglycans, which are preserved after the decellularization process. Studies have indicated that collagen type IV is a key signaling molecule that activates axon fasciculation and promotes axonal growth [32, 33]. LN can enhance the differentiation, migration and adhesion of Schwann cells and following PNI can bind to integrin receptors to facilitate nerve regeneration [1, 34, 35]. The presence of proteoglycans aids axonal regeneration after PNI [36, 37]. Additionally, certain cytokines such as TGF-β were identified, which can influence cell growth, differentiation, apoptosis and immunomodulation through various signaling pathways [38–40]. However, the mechanisms by which the components of the DEP participate in cellular signaling pathways to regulate the repair of injured nerves remain to be further explored.

Through biocompatibility experiments, it has been observed that in addition to being non-toxic to Schwann cells, the DEP promotes their adherence and growth on its surface. This is attributed to the existence of proteins like LN and FN [35, 41] in DEP, which promotes cell adhesion and proliferation. Additionally, through subcutaneous implantation experiments in rats’ backs, it has been demonstrated that the material does not cause severe immune rejection reactions upon contact with tissue. This is due to the substantial removal of cellular antigenic components from the epineurium. Therefore, the DEP could be considered a safe and effective material in the field of neural repair.

Furthermore, in this research, we made a model of sciatic nerve transection in rats and optimized the regrowth of transected peripheral nerves by using the DEP. The study employed the claw-spread reflex test assess the rat sciatic nerve’s functional recovery. This test can show that both motor and sensory nerve function have recovered at the same time. From the experimental results, at the 4-week mark, the claw-spread reflex test showed poor functional recovery in rats, with both material groups showing no significant advantage over the untreated group. This could be because, in the short period after surgery, effective nerve innervation had not yet been established, and the regenerating nerves had not completely reached the target muscles, so the sensory and motor functions of the rats had not been restored. By the eighth week, the groups using the DEP and the DNRM showed a significant advantage in promoting nerve regeneration compared to the untreated group. This demonstrates the effectiveness of using anti-adhesion membranes after end-to-end nerve anastomosis surgery. Additionally, when evaluating the recovery of motor function in tissues innervated by the sciatic nerve, one often used indicator is the SFI [25]. Early on the deficiency of the sciatic nerve, SFI value is very low. As the treatment time increases, this value changes accordingly. An increase in the SFI indicates that the material has a certain reparative effect on the deficient nerve. From the experimental results, there was a significant upward trend in the SFI from the fourth to the eighth week. Notably, in the eighth week, the group with the DEP exhibited the highest SFI value, demonstrating that the DEP promotes the recovery of impairments in the sciatic nerve.

Electrophysiological examination results indicate that both the DEP group and the DNRM exhibited significantly better nerve conduction velocities compared to the untreated group. Notably, at the 4-week mark, as the nerve anastomosis location was exposed again, the DNRM had almost completely degraded, whereas the DEP group had only partially degraded. This partial degradation provided a physical barrier that avoided fibroblasts invading the anastomosis site. Therefore, during the nerve repairment and regeneration, the degradation rate of DEP is more closely matched with the timeline of nerve regeneration, demonstrating a more suitable effect in preventing adhesions.

Toluidine blue staining can be employed to further observe the number of axons, myelin sheath thickness and diameter in the cross-section of the regenerated nerve. It is observable that the diameters and thicknesses of the regenerated nerve axons vary, with a sparse and irregular arrangement. However, in contrast to the other two groups, the group with the DEP showed a more orderly arrangement of regenerated nerves and higher myelin sheath maturity. This was more evident in the eighth week. These morphological changes are consistent with the trends observed in the SFI and electrophysiological results.

The assessment of nerve-associated marker expression through Tuj1 and S-100 immunofluorescence staining on the regenerated nerves provides insights into the degree of nerve regeneration. The level of expression of these nerve-associated markers can reflect the effectiveness of nerve regeneration to some degree. At the 4-week mark, the expression levels of both Tuj1 and S-100 were relatively low, indicating poor nerve regeneration at this stage. By the eighth week, however, there was a significant increase in the expression of Tuj1 and S-100, with the highest expression levels observed in the DEP group. This suggests that the DEP can enhance the expression levels of nerve-associated markers, thereby promoting nerve regeneration.

## Conclusion

The DEP prepared in this study has effectively removed cell-derived antigenic components while preserving a substantial amount of ECM proteins and wrapping the nerve anastomosis site with DEP to achieves the perfect biomimicry. This is the first instance of employing DEP as a novel promising anti-adhesion biofilm for enhancing surgical outcomes of peripheral nerve repair.

## Supplementary Material

rbae054_Supplementary_Data
