# Supplementary material for: Preparation and evaluation of decellularized epineurium as an anti-adhesive biofilm in peripheral nerve repair
Source: Regen Biomater. 2024 May 13;11:rbae054. doi: 10.1093/rb/rbae054 (PMC11153341; doi:10.1093/rb/rbae054)

Supplementary Figure 1

| 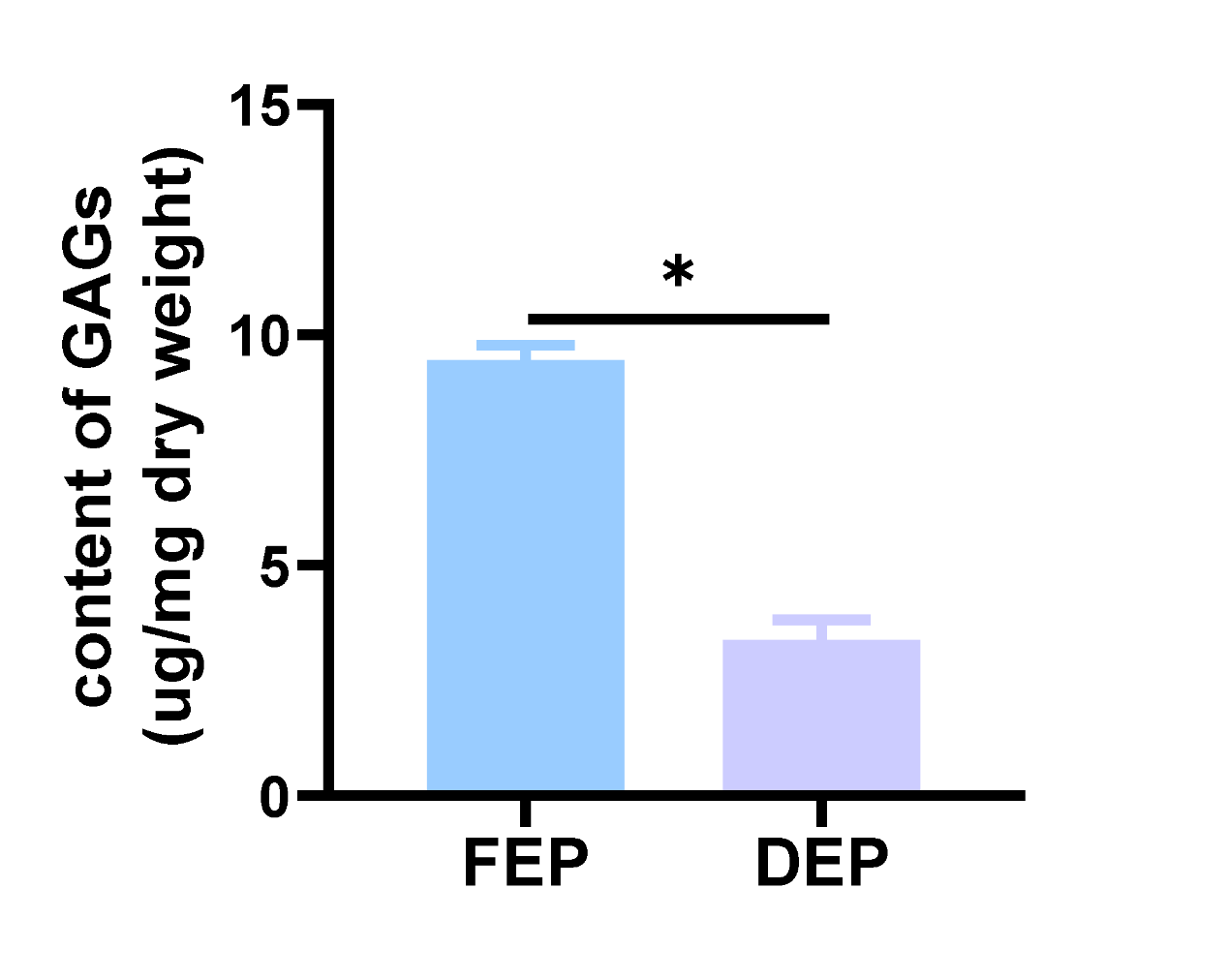 |
| --- |
| **Supplementary Fig. 1** GAGs content. FEP: Fresh epineurium; DEP: Decellularized epineurium. Data are expressed as the mean ± SD (n = 5). **p* < 0.05 |

Supplementary Figure 2

| 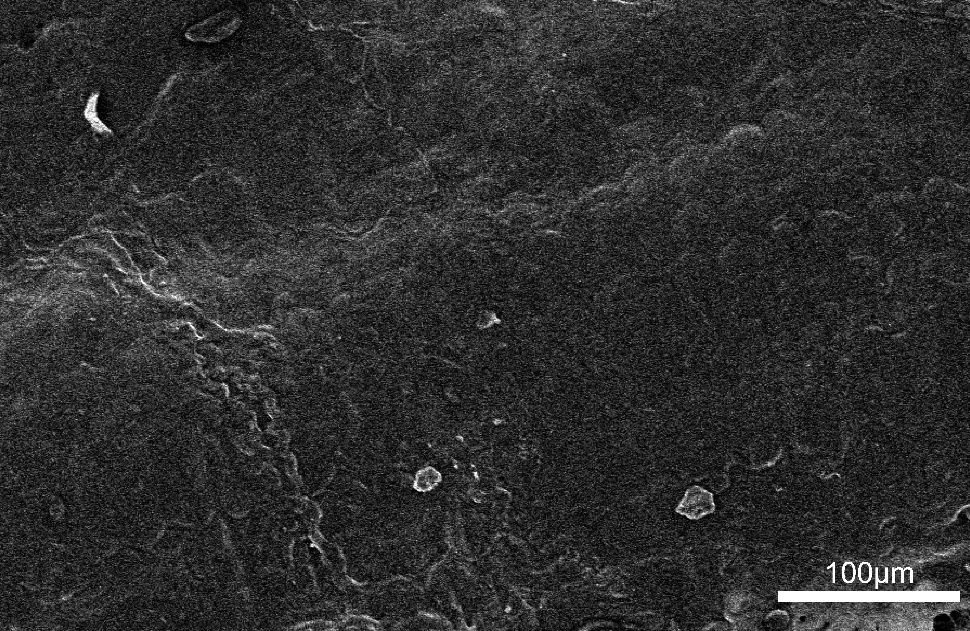 |
| --- |
| **Supplementary Fig. 2** The microstructure of decellularized epineurium is observed by SEM at magnifications of × 200. Scale = 100 μm. |

Supplementary Figure 3

| 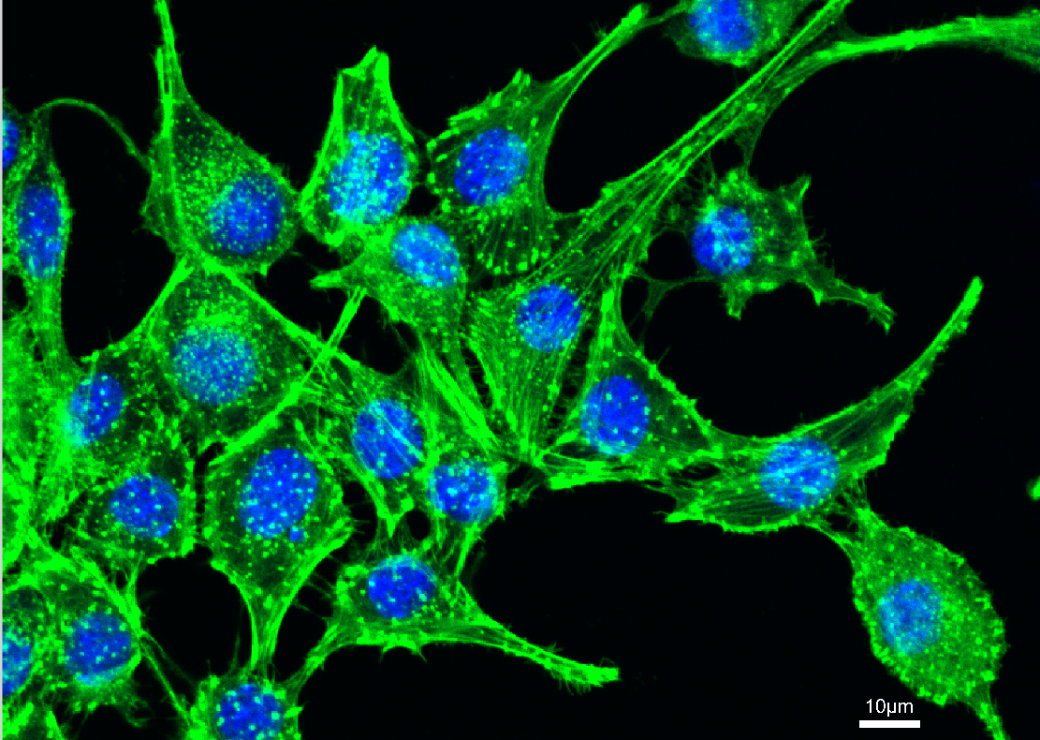 |
| --- |
| **Supplementary Fig. 3** Fluorescence image of RSC 96 stained with F-actin on day 3. Scale = 10 μm |

Supplementary Figure 4

|  |
| --- |
| **Supplementary Fig. 4** Quantification of immunohistochemical staining by positive area (%). Data are expressed as the mean ± SD (n =5) **p* < 0.05. |


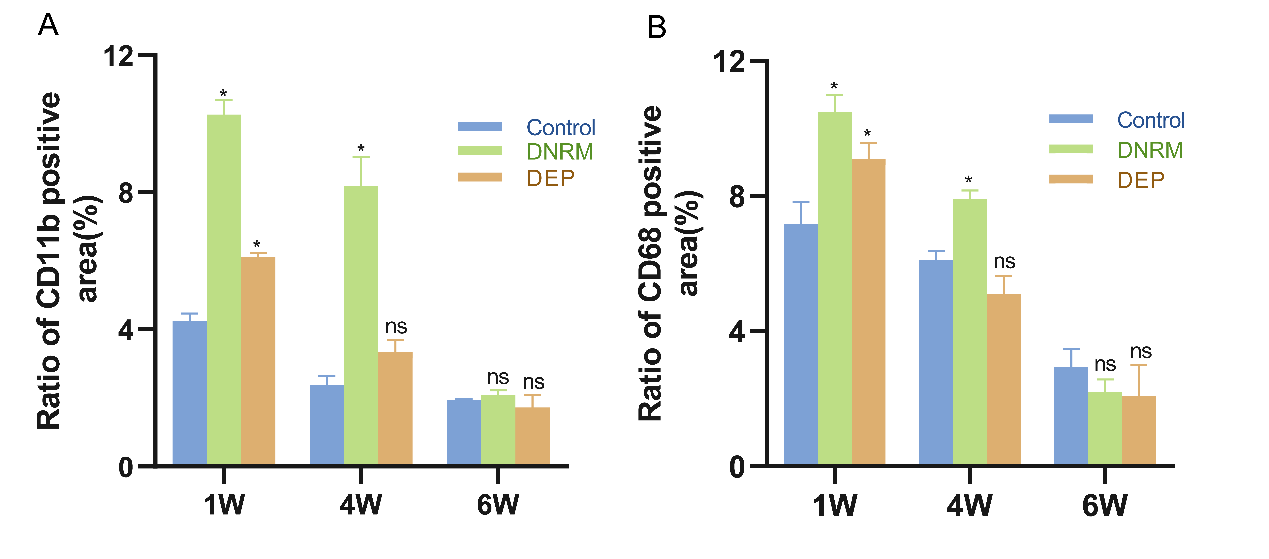

Supplement: rbae054_Supplementary_Data [file rbae054_supplementary_data.docx]
